# Robust Model Selection for Classification of Microarrays

**DOI:** 10.4137/cin.s2704

**Published:** 2009-06-25

**Authors:** Ikumi Suzuki, Takashi Takenouchi, Miki Ohira, Shigeyuki Oba, Shin Ishii

**Affiliations:** 1 Graduate School of Information Science, Nara Institute of Science and Technology, Takayama, Ikoma, Nara 630-0192, Japan; 2 Division of Biochemistry, Chiba Cancer Center Research Institute, Chiba 260-8717, Japan; 3 Graduate School of Informatics, Kyoto University, Gokasho, Uji, Kyoto 611-0011, Japan; 4 PRESTO, Japan Science and Technology Corporation

**Keywords:** gene expression, cancer diagnosis, mini-chip microarrays, supervised analysis

## Abstract

Recently, microarray-based cancer diagnosis systems have been increasingly investigated. However, cost reduction and reliability assurance of such diagnosis systems are still remaing problems in real clinical scenes. To reduce the cost, we need a supervised classifier involving the smallest number of genes, as long as the classifier is sufficiently reliable. To achieve a reliable classifier, we should assess candidate classifiers and select the best one. In the selection process of the best classifier, however, the assessment criterion must involve large variance because of limited number of samples and non-negligible observation noise. Therefore, even if a classifier with a very small number of genes exhibited the smallest leave-one-out cross-validation (LOO) error rate, it would not necessarily be reliable because classifiers based on a small number of genes tend to show large variance. We propose a robust model selection criterion, the min-max criterion, based on a resampling bootstrap simulation to assess the variance of estimation of classification error rates. We applied our assessment framework to four published real gene expression datasets and one synthetic dataset. We found that a state-of-the-art procedure, weighted voting classifiers with LOO criterion, had a non-negligible risk of selecting extremely poor classifiers and, on the other hand, that the new min-max criterion could eliminate that risk. These finding suggests that our criterion presents a safer procedure to design a practical cancer diagnosis system.

## 1. Introduction

Microarray technology^1^ has been applied to predict prognosis of cancer patients by comparing gene expression profiles in cancer tissue samples, and its predictive power has been demonstrated for many types of cancers.^2^–^5^ The prognosis prediction systems based on microarrays have been expected to be new efficient bio-markers that enable personalized cancer medicine.^6^ We consider, in this paper, two problems in expanding the use of microarray-based prediction systems in real clinical scenes, namely, observation cost and reliability.^7^

To reduce the observation cost without losing reliability, there have been several efforts to design diagnosis systems involving small numbers of specially selected genes. Recently, specialized diagnostic microarrays harboring small numbers of genes, to say tens or hundreds genes, are developed based on a supervised analysis with a dataset taken by a full microarray system involving thousands or tens of thousands of genes.^5^,^8^,^9^ Measurement cost per patient becomes smaller by reducing the number of genes that is involved in such a system. If number of spots on a chip is fixed, more spots corresponding to a single gene can be included in a chip, which enables more reliable measurement by averaging multiple spots of same genes, and/or more efficient measurement by diagnosing multiple patients simultaneously in a single chip.^8^ Manufacturing cost of a chip can be reduced by designing mini-chip harboring small number of spots.^5^

To achieve a reliable predictor, a well-known trade-off problem exists even if the above-mentioned issue of observation cost is omitted; we should select as large a number of informative genes and as small a number of non-informative genes as possible. We often need a certain number of genes to gain prediction accuracy, partly because multiple informative genes tend to provide different kinds of information which are complementary to each other for the prediction, and partly because, even when a set of multiple genes provides identical information, observation noise can be reduced by averaging them. On the other hand, since the prediction error increases when non-informative genes are included, we need to reduce the number of non-informative genes, putting the observation cost aside. These two demands are a trade-off because the process of determining whether each gene is informative or non-informative itself is not always reliable enough, due to non-negligible noise and a limited number of observations.

In summary, our goal can be stated as to achieve a reliable predictor based on as few genes as possible, which is accomplished in a supervised analysis with the following three processes:

- a gene selection process,
- a supervised learning process that constructs predictors based on a labeled set of expression data of the selected genes, and
- an assessment process for the constructed candidate predictors.

There have been many options proposed for the first two processes, and comparisons of their combinations were made from the viewpoint of prediction error rates on test datasets, namely generalization performances.^10^,^11^ In the present study, we use the following two procedures that were applied in the previous study.^12^

- Weighted voting (WV) classifier^13^ with gene selection based on absolute t-score (T-WV)
- Linear-kernel support vector machine (SVM)^14^ with recursive elimination of genes that have the smallest contribution to current classification performance (R-SVM).^15^

These procedures construct multiple candidate predictors with various numbers of genes included in the predictors. Since their prediction performances for independent test datasets depend on the number of genes, their assessment is crucial.

In the assessment process, the prediction performance of each candidate predictor is estimated based on the training data, and good estimation is obtained by reducing the estimation bias and the variance. Since the true performance on independent data in the future is unknown, we should select the best predictor with less bias and smaller variance of the estimated performance. In general, the bias-variance trade-off problem is inherent to all statistical models used for prediction, especially in the classification framework.^16^,^17^ For prognosis prediction by microarray, several past studies focused on reducing the estimation biases of the prediction error rates in determining the best model^18^–^20^ because inclusion of biases could lead to over-estimation of the classification performance of the proposed system. The cross-validation (CV) technique is used widely for predicting true classification error rate in samples that are not included in either the training or the test sample sets. Among the CV methods, the leave-one-out cross-validation technique (LOO) is often used because of its small bias.^18^ These studies, however, paid little attention to the variances of estimated classification error rates.

The estimated variances in the assessment process are important for practical applications. Even if a classifier has a sufficiently low error rate accompanied instead by large variance in prediction, it suffers from a high risk of having a large actual error rate when applied to unknown test samples.^21^ The LOO criterion sometimes selects a classifier involving a very small number of genes, or even a single gene. Although the single-gene classifier fits the ‘as few genes as possible’ criterion, classifiers involving redundant genes tend to exhibit lower noise and provide better prognosis.^9^ Several recent methods consider the estimated error rate variances,^21^–^24^ and unsupervised methods^25^,^26^ also minimize the variance of the model by focusing on the stability of the signatures instead of on the supervised class labels. However, there has been no discussion from the viewpoint of mini-chip design, namely, to explore a reliable predictor based on as few genes as possible.

In the present study, we consider both the bias and the variance of performance estimation so as to achieve a reliable predictor. We applied a bootstrap sampling method to estimate the distribution of possible error rates, with bias and variance, and propose a min-max criterion to obtain a stable classifier. We conducted a simulation study and found that the min-max criterion tends to select better candidate predictors than the LOO criterion, especially when the number of samples is small. We then compared two supervised analysis procedures, T-WV and R-SVM, and showed that T-WV achieves reliable predictors with a small number of genes, indicating that T-WV with the min-max criterion is desirable for our purpose of obtaining a reliable predictor with as few genes as possible.

## 2. Methods

### 2.1. Notations

Let *x_i_* = (*x_i_*_1_,..., *x_iM_*) be a vector of the *M*-dimensional gene expression profile of the *i*-th sample, and *y_i_* a binary class label *y_i_* ∈ {−1,1} representing the binary status of the *i*-th sample, for example, tumor or non-tumor. The numbers of samples in the negative ( *y_i_* = −1) and positive (*y_i_* = 1) classes are denoted as *n_n_* and *n_p_*, respectively. Suppose that we have a dataset *D* = {*d_i_*|*i* = 1,..., *N* } including *N* samples, where *d_i_* = (*x_i_*, *y_i_*) is a pair of input (expression) and output (class label) of the *i*-th sample. By applying a supervised machine learning method to the dataset *D*, we construct a discriminant function *h*(*x* | *D*) such that we predict a label *ŷ*(*x*′) for a new input *x*′ by

(1) $$\hat{y}(x^{\prime })=\{\begin{array}{ccc} 1 & \text{if} & h(x^{\prime }|D)\geq 0\\ -1 & \text{if} & h(x^{\prime }|D)<0 \end{array}$$

### 2.2. T-WV method

The WV method is a typical supervised machine learning method that employs the top *k* significant genes. Since the significance of the *j-*th gene is defined according to the following t-score, the entire procedure is referred to as the T-WV method,

(2) $$t_{j}=\frac{{\bar{x}}_{pj}-{\bar{x}}_{nj}}{\sqrt{1/n_{p}+1/n_{n}}S_{j}},$$

where *x̄_pj_* and *x̄_nj_* are the average expression levels of the *j-*th gene over the training samples labeled 1 and −1, respectively, and *S_j_*^2^ is the pooled within-class variance of the *j-*th gene,

(3) $$S_{j}^{2}=\frac{\sum_{i:y_{i}=-1}{\left(x_{ij}-{\bar{x}}_{nj}\right)}^{2}+\sum_{i:y_{i}=1}{\left(x_{ij}-{\bar{x}}_{pj}\right)}^{2}}{n_{n}+n_{p}-2}.$$

The genes are ranked according to the absolute value of *|t_j_|*, and the top-ranked *k* genes are selected as significant genes so that the set of these genes is denoted as *C_k_*. The discriminant function obtained by the T-WV method is then constructed as

(4) $$h_{k}(x|D)=\frac{1}{k}\sum_{j\in C_{k}}t_{j}(x_{j}-{\bar{x}}_{j}),$$

where ${\bar{x}}_{j}\equiv \frac{1}{N}\Sigma_{j}^{N}x_{ij}$ is the average expression level of the *j-*th gene in the training samples.

In the discriminant function *h_k_*, the difference between the *j-*th gene expression and its average is weighted by its significance, i.e. the t-score. Note that the function *h_k_* depends on the number *k* of significant genes, and thus we need to specify *k* appropriately.

### 2.3. R-SVM method

R-SVM is another typical supervised machine learning method, which was developed to select important genes for SVM classification.^15^ An R code package is publicly available at http://www.hsph.harvard.edu/bioinfocore/R-SVM.html. The discriminant function of a linear SVM is defined as

(5) $$h_{k}(x^{\prime }|D)=(w\cdot x^{\prime })+b=\sum_{i=1}^{N}\alpha_{i}y_{i}(x_{i}\cdot x^{\prime })+b,$$

where *x′* is a new input expression vector and *x_i_* is the *i*-th sample expression vector in the training dataset. *α_i_* and *b* are parameters to be determined so that training data points with different class labels are classified with the largest margin. *x* · *x*′ = ∑*_j_*_=1_*^M^* *x_j_* *x*′*_j_* denotes the inner product. Each element of *w*, *w_j_*, is defined as

(6) $$w_{j}=\sum_{i=1}^{n}\alpha_{i}y_{i}x_{ij},$$

the absolute value |*w_j_*| of which represents the significance weight of the *j*th gene in the current discriminant function.

As in the T-WV method, the classification performance of SVM also depends on gene subset selection. R-SVM applies a recursive feature elimination (RFE) procedure.^27^ In RFE, less significant genes in the current discriminant function are recursively eliminated, and the next discriminant function is constructed based on the new, smaller set of genes. Consequently, a sequence of discriminant functions with decreasing numbers of genes is constructed. Thus, the prediction performance of each discriminant function *h_k_* depends on the number *k* of significant genes, which causes the same problem as in T-WV, i.e. setting an appropriate number *k.* In the following section, we describe a common way to set the number of genes in both T-WV and R-SVM.

### 2.4. LOO model selection

T-WV and R-SVM, both produce many candidate classifiers, from which we should select the best one by an assessment process. Although the true performance of a classifier is measured as classification accuracy on an unknown dataset given in the future, we should instead estimate the performance using the dataset obtained in the assessment process. Note that we refer to each candidate in the assessment process as a *model*, to clarify that we are assessing all procedures used to construct a classifier rather than assessing solely the classifier. In T-WV and R-SVM, a model is characterized by the number of significant genes that it includes.

The LOO procedure has been widely used to estimate, or predict, the future performance of a classifier. In LOO, a classifier *h* is built using each leave-one-out dataset *D*^−^*^i^*, *i* = 1,..., *N*; that is, the *i*-th sample *d_i_* is excluded in the training procedure from the dataset *D*, and becomes a validation sample. The classification performance of *h* is assessed using the validation sample. After the assessments for *d*_1_,..., *d_N_*, the LOO error rate of the classifier *h*, *G*_LOO_(*h* | *D*), is calculated as the averaged error rate

(7) $$G_{\text{LOO}}(h|D)=\frac{1}{N}\sum_{i=1}^{N}I(y_{i}h(x_{i}|D^{-i})<0),$$

where *I*(*R*) denotes the indicator function that takes a value of one if condition *R* holds, and is otherwise zero. When we select the number *k* of significant genes by

(8) $$h_{k}^{\text{LOO}}=\underset{k}{\text{argmin}}G_{\text{LOO}}(h_{k}|D),$$

this model selection is said to be based on the LOO criterion.

### 2.5. Resampling bootstrap method

It is known that the error rates used to estimate the LOO procedure are nearly unbiased. Molinaro et al^18^ compared estimated generalization error rates between different resampling methods and showed that LOO had the smallest bias for a simulation dataset and a real microarray dataset. However, LOO has a tendency to include large variance, despite its small bias,^28^ because classifiers constructed based on the leave-one-out datasets, *D*^−^*^i^*, are quite similar to each other, whereas the data points used for validation vary widely. The large variance of the error rate estimation leads to a high risk of selecting a classifier whose ‘true’ performance is poor, and this risk becomes higher as the number of candidates becomes larger. When we assess the performance of many candidate classifiers with large variances, some of the candidates often exhibit remarkably low errors, even if their true performance is poor. This is the same problem as overfitting, which was originally found in parametric learning especially when there are many parameters to be learnt. Therefore, it is important to reduce the estimation variance to obtain a robust classifier.

We applied a bootstrap method to simulate possible variation of the given dataset and to obtain the distribution of LOO error rates over the range of that variation. We generated bootstrap datasets {D*^b^ *| b* = 1,..., *B*}, in which each bootstrap dataset is defined as

(9) $$D^{*b}=\{d_{r}^{*b}=(x_{r}^{*b},y_{r}^{*b})|r=1,\ldots ,N-1\},$$

where d_r_^*^^b^ is randomly sampled with replacements from the LOO dataset *D*^−^*^i^*. The single validation sample *d_i_* is evaluated by the classifiers that were trained by different datasets D*^b^, leading to a set of LOO error rates: *G*_LOO_(h_k_^*1^|D^*1^), *G*_LOO_(h_k_^*2^|D^*2^),..., *G*_LOO_(h_k_^*^^B^|D^*^^B^). h_k_^*^^b^, *b* = 1,..., *B*, is given by Eq. (4) after replacing the dataset *D* with the bootstrap dataset D*^b^. This set of LOO error rates is considered to be a distribution of *G*_LOO_ and provides a guideline to determine the number of genes used in the T-WV classifier.

### 2.6. Min-max model selection

Using the simulated distribution of LOO error rates, {*G*_LOO_(h_k_^*^^b^|D^*^^b^)}*_b_* _=1_*^B^*, we defined a risk score called a min-max criterion,

(10) $$G_{\text{BOOT}}(h_{k}|D)=\text{Per}95({\left\{G_{\text{LOO}}(h_{k}^{*b}|D^{*b})\right\}}_{b=1}^{B}),$$

where ‘Per95’ denotes the 95th percentile of the set of values. Based on this risk score, an appropriate model (i.e. the number of genes, *k*) is selected as

(11) $$h_{k}^{\text{BOOT}}=\underset{k}{\text{argmin}}\{G_{\text{BOOT}}(h_{k}|D)\}.$$

We considered the 95th percentile with the number of bootstrap *B* = 100 as the representative of possible high error rates for each model with different numbers of genes. The 95th percentile is a robust criterion to estimate the risk of selecting a bad model against the possibly asymmetric nature of the error rate distribution.

Our approach is referred to as the “min-max” selection criterion because we minimized the risk of selecting a model for which the expected prediction error rate was almost the maximum in the distribution of possibilities. This min-max model selection is likely to refuse classifiers for which the estimated error rates are distributed with a large variance, even if LOO shows the lowest error rate from a single dataset. Therefore, the min-max criterion reduces the instability stemming from the variation of possible future datasets that could be simulated by random sampling from a large pool of samples.

In other words, the min-max criterion assumes an underlying game between an analyzer and nature. A dataset is given by nature, and a model is selected by an analyzer. For the analyzer to achieve stability, one good idea is to minimize the risk (Eq. (11)), which stems from the possibility that nature could provide a bad situation (and hence the classifier has been over-trained) (Eq. 10).

The number 95 of the percentile and number of bootstrap *B* = 100 were determined arbitrarily by considering trade-offs between computation time, estimation variance of the percentile point, and appropriateness as a representative of high error rates:

- The computation time is proportional to the number of bootstrappings.
- Estimation variance is a monotonic function of both the percentile number and the number of bootstrappings. Namely, the variance becomes large as the percentile number diverges from 50 and as the number of bootstrappings is small.
- The criterion should evaluate possible high error rates even when the distribution of bootstrap samples is asymmetric.

We did not select the 50th percentile, i.e. the median, because of the third reason above; we attempted to obtain a safe classifier rather than to show good average performance. Although the 99th percentile could be another representative of possible high error rates, we rejected it, because it relies on 1% of bootstrap samples, and will therefore lead to high variance especially with small *B.* The estimation variance of each percentile of the bootstrap error rate can be evaluated in terms of the standard deviation of the corresponding order statistic if the distribution of error rates is known. Table 1 shows the standard deviations (SDs) of several percentiles when the distribution of error rates is a standard normal distribution. These SDs are proportional to the SD of the distribution of error rates, implying that the SDs of the percentiles can represent their variation well even for non-normal distributions.

## 3. Results

### 3.1. Results for real datasets

We evaluated our method using four published real gene expression profile datasets:

- Breast cancer
  van’t Veer et al^3^ obtained gene expression microarray data for approximately 5,000 genes for 78 + 19 breast cancer tissue samples. The samples were classified into favorable and unfavorable samples: patients with recurrence-free survival in five years and those with recurrence in five years, respectively. The authors trained supervised classifiers using 78 samples (34 favorable and 44 unfavorable samples), which we call the training dataset, and tested using 19 independent samples (7 favorable and 12 unfavorable samples), which we call the test dataset. The same group also provided a larger dataset consisting of 295 samples.^29^ Among the 295 samples, 32 samples were also included in the former dataset^3^ and 10 samples were censored in five years; hence, we used the remaining 253 (192 favorable and 61 unfavorable) samples for the second test dataset.
- Colon cancer
  The colon cancer dataset^30^ contains microarray expression data for 2,000 genes for 62 colon tissues. Among the 62 tissue samples, 40 and 22 were labeled as “tumor” and “normal,” respectively, and these were used as the labels to be predicted.
- Neuroblastoma (NBL)
  The NBL dataset^5^ consists of microarray expression data for 5,180 genes for 136 patients. Among the 136 samples, 25 and 102 were labeled as “favorable” and “unfavorable” patients, respectively, according to their status at 24 months after diagnosis, and these were used as the labels to be predicted. The remaining nine samples of unknown status at 24 months after diagnosis were omitted.
- Breast cancer Affymetrix (Affymetrix)
  Wang et al^31^ analyzed 286 breast cancer patients with an Affymetrix chip harboring 22,283 genes. Among the 286 patients, 183 and 93 were labeled as favorable and unfavorable, respectively, and these were used as the labels to be predicted. We omitted 10 samples which were censored in five years. Although this dataset concerned breast cancer, we did not consider relationship between this set and the breast cancer datasets at the top of this list because these two datasets were assembled by entirely different systems and hence had fairly different characters in distribution. Considering different systems of microarrays together may be an important issue, but is beyond the scope of the current study.

For each of the above four datasets, we trained T-WV and R-SVM classifiers with various numbers of genes using the training samples, and assessed their classification errors in terms of LOO, 3-, 5- and 10-fold-CV, and min-max criteria. In the case of the breast cancer dataset with large numbers of test samples,^3^,^29^ we also assessed their classification errors in the test datasets.

**Figure 1:**
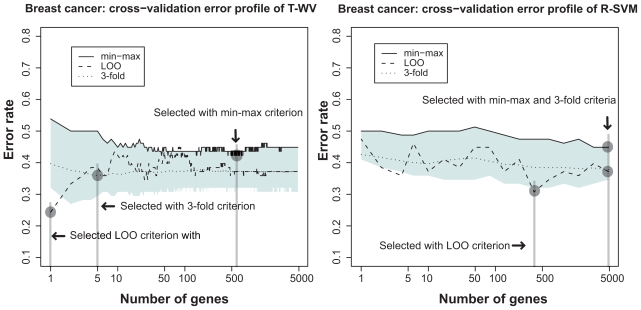
Estimated classification errors in the breast cancer dataset. The left and right panels show the results obtained with the T-WV and R-SVM methods, respectively. The vertical and horizontal axes denote classification error rates estimated by various criteria and the number of genes included in each classifier, respectively. The 90% interval of resampling bootstrap of the estimated classification errors at each number of genes is denoted by blue areas. The classification errors estimated by the three criteria, min-max criterion (solid line on the top of blue area), LOO error rate (dashed line), and 3-fold-CV error rate (dotted line), are plotted against different numbers of genes. Vertical lines indicate the numbers of genes selected by the three criteria.

Figure 1 shows the results for the breast cancer dataset. The results with the T-WV classifier (left panel), indicated characteristic behaviors of the three criteria to assess the classification error rate, LOO (dashed line), 3-fold-CV (dotted line), and the proposed min-max criterion (solid line at the top of the blue area). The 90% interval of LOO error rates (blue area), which was estimated by the resampling bootstrap method, describes the estimation variance of error rates. The LOO error rate profile showed the lowest value with a small number of genes, *k* = 1, so that *k* = 1 was selected as the best number of genes by the LOO criterion. On the other hand, the 90% interval of the bootstrap distribution at *k* = 1 exhibited a large width in the error rate, and the 95th percentile error rate was above the chance level 0.5, suggesting large risk of the *k* = 1 classifier falling into a poor predictor around the chance level. Also, the LOO error rate at *k* = 1 was below both the 5th percentile and the 3-fold-CV error rate, indicating that the low LOO error rate at *k* = 1 could have been obtained by chance. The 3-fold-CV showed a smoother profile than those obtained by the LOO, and stayed in the midst of the 90% interval. The 3-fold-CV criterion selected a classifier with *k* = 5 where the 90% interval was narrower than that at *k* = 1. We also calculated 5- and 10-fold-CVs and observed similar curves to that of the 3-fold-CV. The proposed min-max criterion, i.e. the 95th percentile, selected a larger number of genes, *k* = 590. The LOO and 3-fold-CV error rates at *k* = 590 were higher than those at *k* = 1 and *k* = 5; however, we expected that the classifier of *k* = 590 would have a lower risk of being a poor predictor than those at *k* = 1 and *k* = 5.

In the right panel of Figure 1, a similar comparison is shown between LOO, 3-fold-CV, and the min-max criteria with the R-SVM classifier. The LOO criterion showed an instability similar to that of T-WV, so that the lowest LOO error rate at *k* = 376 seems to have been obtained by chance. All criteria selected larger numbers of genes than in the cases of T-WV classifiers.

In Table 2, test error rates of the selected predictors were assessed using two test datasets with 19 and 253 samples, where five criteria (LOO, min-max, and 3-, 5- and 10-fold-CVs) with two classifiers (T-WV and R-SVM) are compared. The min-max criterion outperformed the other criteria, LOO and k-fold-CVs, on both test sets. The LOO exhibited poor performance with 19 test samples and worse with 253 test samples whose test error rate was around the chance level. Intuitively, this result pointed out a defect of the LOO criterion in terms of the risk of taking a poor classifier, which has already been suggested by the 90% interval shown in Figure 1. The 3-, 5- and 10-fold-CVs achieved better performance in test error rates than LOO, but worse than the min-max criterion. T-WV tended to exhibit lower error rates than R-SVM with smaller numbers of genes, although we cannot conclude the general superiority of T-WV based on this single example.

Test error rates on 253 samples were significantly worse than the error rates on 19 samples, possibly for the following reasons:

- The 19 samples were by themselves easily classified.
- The number of samples (19) was too small to reproduce the error rate with low variance.
- The test data of 253 samples were gathered from different populations from those for the training data of 78 samples and the other test data of 19 samples.
- The microarray measurement system differed between the two sets of data.

The considerations above will be important when designing mini-chips based on training datasets. Although the last reason, difference in microarray systems, may not be very serious in the case of this breast cancer dataset, it would be serious in the case designing a mini-chip, because differences between systems will probably be inevitable due to the reduction of system size from a full-size chip to a mini-chip.

**Figure 2:**
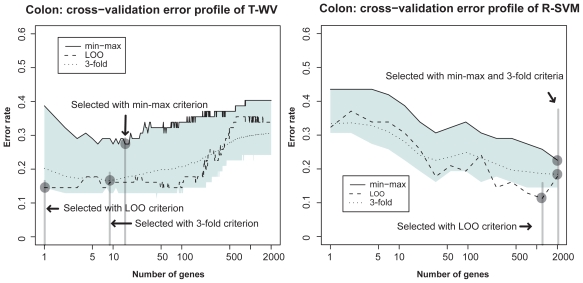
Estimated classification errors in the colon cancer dataset. See Figure 1 legend for details.

**Figure 3:**
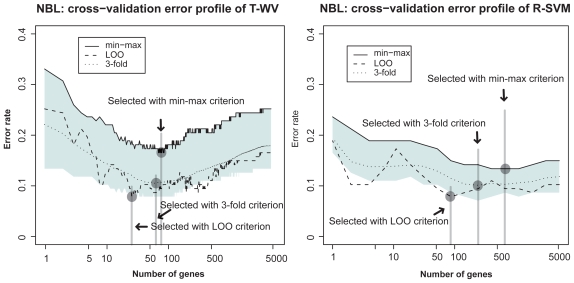
Estimated classification errors in the NBL dataset. See Figure 1 legend for details.

**Figure 4:**
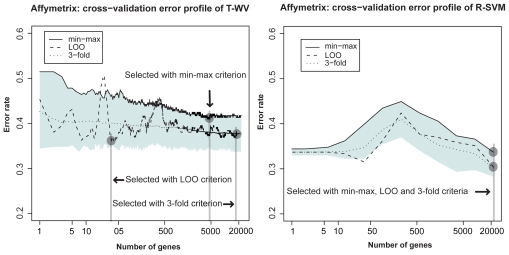
Estimated classification errors in the Affymetrix dataset. See Figure 1 legend for details. Note that the errors of R-SVM with *k* < 10 are not reliable because of unbalanced numbers of labeled samples.

We compared three criteria, LOO, min-max, and 3-fold-CV, with the two classifiers T-WV and R-SVM on the other three datasets (NBL, colon cancer and breast cancer Affymetrix) in Figures 2, 3 and 4, respectively. From the total comparisons over Figures 1–4, we observed the following tendencies:

- Although the error rates estimated by LOO fluctuate as the number of genes increases, they stay mostly within the 90% interval. This suggests that the LOO estimation of the tuned number of genes includes a large variance and the character of the variance is well captured by the estimated 90% interval.
- In contrast to the fluctuating profile of LOO error rates, the profiles of the 3-fold-CV and the 95th percentile (*G*_BOOT_) exhibit smoother curves. This suggests a more stable character for the 3-fold-CV and the min-max criterion than the LOO criterion.
- With T-WV, the 90% confidence interval was likely to be wide when the number of genes was small, *k* < 10, indicating that prediction based on too few genes is risky; we occasionally get a model with poor performance. The 95th percentile is likely to show a higher error rate for a smaller number of genes, e.g. *k* < 10, than for a large number of genes. Thus, the min-max criterion based on the 95th percentile can avoid risky prediction so that a smaller error rate is achieved on average.
- The 3-fold-CV profile stayed almost in the middle of the 90% interval and showed a similar curve to the 95th percentile. However, there was difference between the 3-fold-CV and the 95th percentile in the range of 90% interval, which was prominent in T-WV with small numbers of genes, *k* < 10. The 3-fold-CV and the min-max criterion lead to different numbers of genes being selected; relatively large numbers of genes are selected by the min-max criterion in comparison to the 3-fold-CV.
- In the case of T-WV, the 90% interval was likely to be narrow for datasets with large sample sizes. The numbers of training samples were 78, 62, 127 and 276, and the widths of the 90% interval were about 0.15, 0.15, 0.1 and 0.07, for breast cancer, colon, NBL and Affymetrix datasets, respectively.
- In the case of R-SVM, LOO profiles fluctuated more than those of the min-max criterion, as well as with T-WV, suggesting that the min-max is a better model selection criterion than the LOO criterion.
- Whereas the best performance was comparable between R-SVM and T-WV, a larger number of genes was required to achieve the best performance by R-SVM than by T-WV. Thus, T-WV employing a relatively small number of genes is more suitable for practical clinical applications, which is consistent with a previous finding.^12^
- The confidence intervals for R-SVM were likely to be narrower than those for T-WV, implying that SVM, as a large margin classifier, is more stable against observation noise than T-WV. Even though we are not interested here in classifiers with a large number of genes, say *k* > 1,000, this finding may be important for applications other than mini-chip construction.
- The Affymetrix data set was unbalanced, with the numbers of favorable and unfavorable samples being 183 and 93, respectively. This suggests that the error rate would become 0.34 if every label prediction is called favorable, which actually occurred for R-SVM with *k* < 10. Therefore, the narrow confidence interval in such a case did not correspond with stable prediction.

The experiments showed that a reduction of risk is achieved by the proposed min-max criterion, and this was particularly convincing in the breast cancer dataset.

### 3.2. Simulation study on synthetic datasets

In the previous section, we tested our new criterion on four real datasets; however, the ground truth was unknown and the number of samples was limited in many cases, which prevented us from obtaining strong evidence for the superiority of the min-max criterion. We conducted a simulation study based on artificial datasets to prepare a sufficient number of test samples, which will be more realistic in future clinical studies.

We randomly generated expression profiles for 2,000 genes, where 30 out of the 2,000 were differentially expressed (DE) between two classes of samples and the others were not (non-DE). For non-DE genes, expression levels were generated from a normal distribution with mean zero, *N*(0,1), and for DE genes, the expression levels of samples with positive and negative class labels were generated from *N*(*μ*, 1) and *N*(−*μ*, 1), respectively, where we set *μ* = 0.5 for all DE genes. By this process, we generated synthetic datasets of 20 to 150 samples for training, and 1,000 samples for testing, where the numbers of samples with the two class labels were set to be equal.

**Figure 5:**
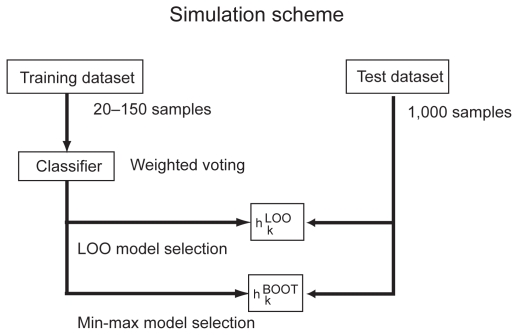
Setting of the simulation experiment.

The proposed simulation scheme is illustrated in Figure 5. For each training dataset, the candidate classifiers involving various numbers of genes were trained and assessed, and the best numbers of genes were selected by the LOO and the min-max criteria, where the number *B* of the bootstrap in the min-max procedure was set at 100. The performance of the finally selected classifier was then assessed by a test dataset with 1,000 samples. We repeated this process with a randomly generated training dataset and assessed the corresponding test error rates by using a test dataset of 1,000 samples. The distributions of the test error rates were compared between different conditions.

We designed the above setting to clarify how well the min-max criterion improves the model selection. The number of test datasets was set sufficiently large, and is commonly used in various settings of the other features to reduce the variance of error rates that stems from random sampling of the test dataset. The number of DE genes (30) and the strength of differential expression (*μ* = 0.5) were determined to examine typical situations that arise in realistic cases. We omitted other realistic features of datasets that may arise such as variation in the number of DE genes, strength *μ*, and the proportion of numbers of positive and negative samples, because they had shown no significant effect in our preliminary experiments. We also omitted correlations of gene expression patterns between DE genes because such correlations would not affect either T-WV or R-SVM.

**Figure 6:**
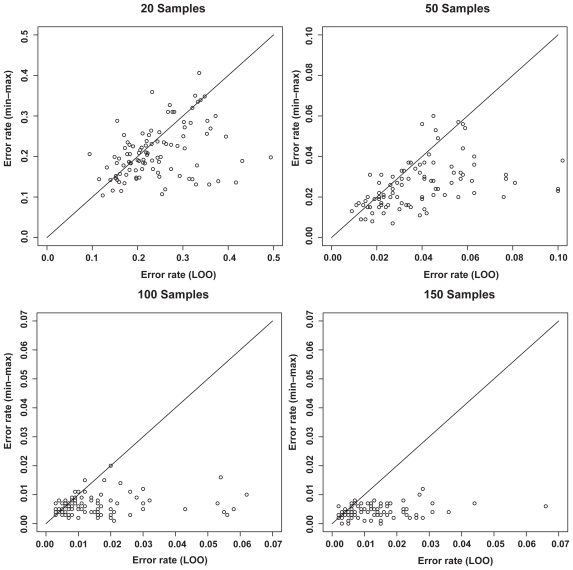
Distribution of test error rates of T-WV. The vertical and horizontal axes denote the test error rates of classifiers selected by the min-max and LOO criteria, respectively. The results from 100 trials of random sampling of 20, 50, 100 and 150 samples are shown in the four panels.

Figure 6 shows the distributions of test error rates of the T-WV classifiers selected by LOO and min-max, with 20, 50, 100 and 150 training samples. We found that there were certain levels of variance for both criteria, and the variance was larger for smaller numbers of samples. LOO sometimes showed much worse results than min-max, as indicated by the points in the bottom-right area of each panel in Figure 6. Note that the number of test samples, 1,000, was so large that there was no significant increase in sampling variance. Table 3 shows the means and standard deviations of test error rates of the classifiers selected by LOO and min-max. Through 20–150 training samples, min-max outperforms LOO in terms of smaller means and smaller standard deviations of test error rates.

We counted the number of true DE genes in the selected genes for each trial and found that the min-max criterion tended to include many of the 30 true DE genes, and that the ratio of the true DE genes in the selected genes became large as the training samples increased. In contrast, LOO sometimes selected a very small number of genes, leading to large error rates. Both criteria occasionally selected more than 30 genes, although this did not cause a large increase in the error if the selected genes included many of the true DE genes. As the number of training samples increased, the means and variances of test error rates became smaller, which is consistent with the previous observation. Even when the number of training samples increased and mean error rates decreased, however, the test error rates of LOO still showed larger variance than those of min-max.

**Figure 7:**
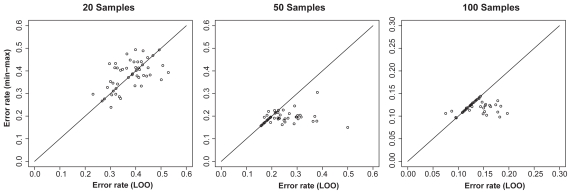
Distribution of test error rates of R-SVM. The vertical and horizontal axes denote the test error rates of classifiers selected by the min-max and LOO criteria, respectively. The results from 50 trials of random sampling of 20, 50 and 100 samples are shown in the three panels.

We also conducted a similar simulation with R-SVM; the simulation settings were the same as those for T-WV except that we performed 50 trials, (half the number used for T-WV), and we excluded the case of 150 samples because of the large computational cost of bootstrap simulation for R-SVM. Figure 7 shows the distributions of test error rates of R-SVM classifiers selected by LOO and min-max with 20, 50, and 100 training samples. A similar tendency to that of T-WV was observed in the cases of 50 and 100 samples, although in the case of 20 samples, the error rate was almost the chance level (0.5) for both the LOO and min-max criteria.

## 4. Concluding Remarks

In the present study, we investigated model selection methods with the aim of designing a reliable cancer prognosis predictor based on gene expression microarrays involving as small a number of genes as possible. We assessed possible variation in prediction error rate of each microarray-based predictor by simulating a distribution of classification error rates via a resampling bootstrap method. Accordingly, we proposed a novel min-max criterion to select a predictor from multiple candidates. In numerical comparisons that used real and synthetic datasets, we showed that the conventional LOO estimation of their error rates resulted in large variances; consequently, the LOO criterion had a large risk of choosing inappropriate classifiers that would exhibit extremely poor prediction performance. In contrast, we showed the stability of the min-max criterion relative to well-established statistical criteria including the LOO. We also compared two different supervised analysis procedures, T-WV and R-SVM, and found that, in general, T-WV performed the best when it involved a small or moderate number of genes in contrast to that R-SVM performed the best when it involved almost all genes, although the mean and variance of the best possible performances were not always significantly different between those achieved with T-WV and R-SVM. Thus, overall, we concluded to recommend T-WV with the min-max criterion, which satisfied our demand; the most reliable predictor involving as small a number of genes.

It should be important to note that, we proposed our procedure to select a set of genes for designing a good predictor of cancer prognosis, rather than for determining a set of genes which have statistically significant relationship to the prognosis; these purposes are different from each other in general. In other words, the ‘robust’ model selection is meant to lower the risk to select an extremely poor predictor, rather than to select a stable set of genes. In fact, different research groups reported prognosis prediction systems with different sets of genes based on different sets of microarray data for the same type of cancer.^6^ The microarray-based predictors for breast cancer, were designed with 70 and 76 genes by two different research groups,^3^,^31^ respectively, and these gene sets had only three genes in common. Namely, the selected sets of genes were not stable at all, however, the 70 gene-based diagnosis system of breast cancer have been verified by increasingly large number of new patients and authorized by Food and Drug Administration in USA.^6^ In our own numerical experiments, we also observed that number of common genes tended to be small between any gene sets that were selected based on different datasets generated by resampling bootstrap (data not shown), although we achieved good predictors in vast amount of the cases as we had shown. Thus, it should be emphasized that such an instable selection of gene subsets did not necessarily cause a poor predictor as long as the predictor was selected by a robust model selection method.

Once a prediction system based on a small number of genes is developed, the system can be transfered not only to mini-chip microarrays but also to other easy accessible devises such as quantitative real-time polymerase-chain-reaction (RT-PCR) analysis,^32^ which would be tractable if only tens of genes were targeted. Robust model selection methods, like the proposed one, will be needed especially when we consider such a transfer work between different measurement devises because large bias is often expected between different devises. In general, when a procedure is designed to be robust against measurement variance, such a method is also robust against an unknown bias which would appear like in the above transfer; thus, our min-max criterion will be used for this purpose.

In order to design a practical tool for real scenes in clinical cancer therapy, new demands in informatics can always arise. As we had seen in this study, although past efforts in informatics tended to pursue good performances in average, minimizing risk to catch poor predictor against possible variability in cancer diagnosis systems becomes a next issue. There are few methods to directly seek such risk minimization as long as we know. Reducing cost by selecting relevant genes based on high-dimensional gene expression profile is a relatively well-investigated field of research. However, the combination of the cost and reliability is not investigated well. Thus, there must be room to develop a novel supervised classification method that satisfies these demands for designing mini-chip systems, and future studies in cancer informatics should proceed to such directions.

## Supplementary Data

**Figure S1:**
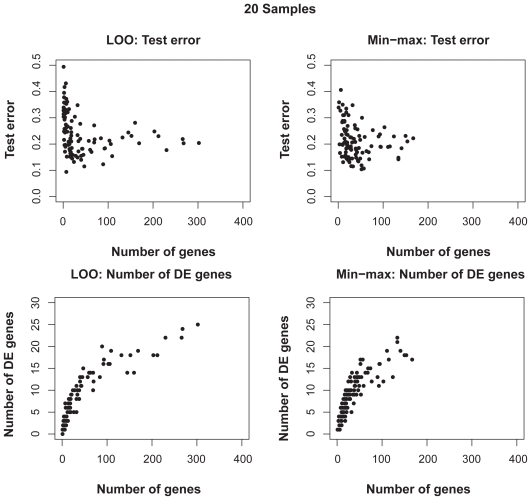
Distributions of test errors and numbers of selected true DE genes for various numbers of selected genes for T-WV classifiers based on 20 artificial samples. Each point denotes one of 100 trials in each setting. Horizontal axes denote the number of genes selected by either LOO or min-max criterion. The vertical axes in the top two panels and the bottom two panels denote the test error estimated by 1000 test samples and the number of true DE genes in the selected set of genes, respectively.

**Figure S2:**
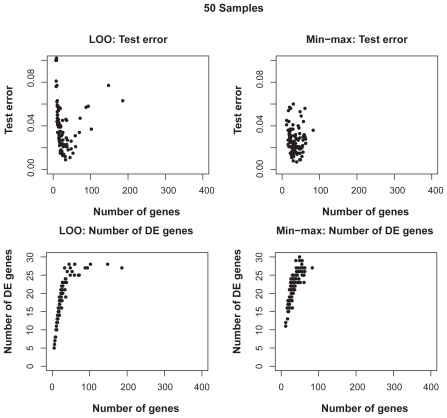
Distributions of test errors and numbers of selected true DE genes for various numbers of selected genes for T-WV classifiers based on 50 artificial samples

**Figure S3:**
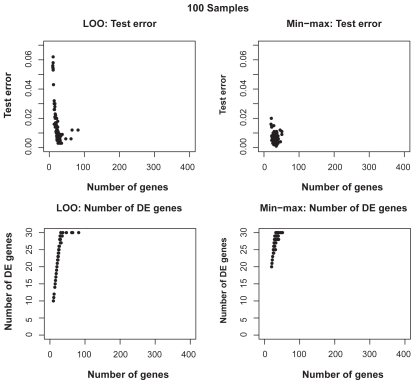
Distributions of test errors and number of selected true DE genes for various numbers of selected genes for T-WV classifiers based on 100 artificial samples.

**Figure S4:**
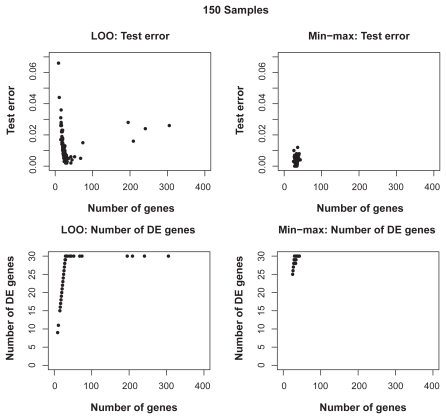
Distributions of test errors and number of selected true DE genes for various numbers of selected genes for T-WV classifiers based on 150 artificial samples.

## Figures and Tables

**Table 1:** Estimated standard deviations of bootstrap percentiles. Bold type marks the setting which we used in the current study.

	*B* = 100	*B* = 500	*B* = 1000
99th	0.315	0.171	0.120
95th	**0.216**	0.095	0.067
90th	0.172	0.077	0.054
50th	0.125	0.056	0.040

**Table 2:** Selected numbers of genes and corresponding test error rates in the breast cancer dataset with LOO, min-max, and k-fold CVs assessed by two test datasets with 19 and 253 test samples.

	T-WV			R-SVM		
	# Genes	Test 19	Test 253	# Genes	Test 19	Test 253
LOO	1	0.2105	0.4862	376	0.4737	0.4664
min-max	590	0.1578	0.2925	4,833	0.4211	0.3992
3-fold	5	0.3158	0.3992	4,833	0.4211	0.3992
5-fold	2	0.2632	0.4071	626	0.6316	0.5217
10-fold	1	0.2105	0.4862	376	0.4737	0.4664

**Table 3:** Test error rate of simulation dataset.

Number of training samples	Selection criterion	Mean	Standard deviation
20	LOO	0.241	0.077
	min-max	0.210	0.064
50	LOO	0.042	0.024
	min-max	0.026	0.012
100	LOO	0.015	0.013
	min-max	0.006	0.003
150	LOO	0.012	0.010
	min-max	0.004	0.002
